# Microvascular density and vascular endothelial growth factor and osteopontin expression during the implantation window in a controlled ovarian hyperstimulation rat model

**DOI:** 10.3892/etm.2015.2181

**Published:** 2015-01-14

**Authors:** XIN GONG, QING TONG, ZHENZHEN CHEN, YUNNA ZHANG, CAI XU, ZHE JIN

**Affiliations:** 1 Reproductive Endocrinology Center, Dongfang Hospital of Beijing University of Chinese Medicine, Beijing 100078, P.R. China; 2 School of Chinese Materia Medica, Beijing University of Chinese Medicine, Beijing 100102, P.R. China

**Keywords:** angiogenesis, adhesion, animal model, Chinese herbs, receptivity

## Abstract

Vascular endothelial growth factor (VEGF) and osteopontin (OPN) are suggested to facilitate angiogenesis and vascular remodeling in endometrial receptivity. Determination of the endometrial microvascular density (MVD) is the commonest method used to indirectly assess the levels of vasculogenesis and angiogenesis; however, the associations among VEGF, OPN and MVD remain unclear. Controlled ovarian hyperstimulation (COH) with the gonadotrophin-releasing hormone agonist-long protocol may impair endometrial receptivity, and Traditional Chinese Medicine (TCM) may exert therapeutic effects to relieve this impairment. The aim of the present study was to investigate the effects of COH on implantation biology and pregnancy outcome, and to explore the potential therapeutic role of the TCM Zi Dan Yin (ZDY). Female Sprague Dawley rats were divided into four groups: Control, COH, ZDY and COH + ZDY. On days 3, 4 and 5 of pregnancy (D3, D4 and D5, respectively), endometrial MVD was measured with cluster of differentiation 34 immunohistochemical detection, and VEGF and OPN protein and mRNA expression was detected through western blotting and reverse transcription-quantitative polymerase chain reaction (RT-qPCR) analysis. On D10, the average number of implantation sites was observed. Subsequent to conceiving and bearing newborn rats, the number of live births from each group was recorded. COH was shown to have adverse effects on implantation and pregnancy outcome. The MVD was found to be significantly increased in the COH group compared with that in the control, ZDY and COH+ZDY groups. The results of the protein and RT-qPCR analysis of VEGF and OPN revealed the same trend. Conversely, ZDY reversed the changes in endometrial MVD, VEGF and OPN, and was indicated to improve uterine receptivity and pregnancy outcome. No significant difference was observed among the control, ZDY and COH + ZDY groups. In conclusion, since the results for MVD and VEGF and OPN expression were consistent, MVD could be used as an alternative approach to identify the period of receptivity in rats.

## Introduction

The implantation of a mammalian embryo is a crucial step in the establishment of a normal pregnancy. To prepare for implantation, the uterus undergoes dynamic and tightly regulated proliferation and differentiation following stimulation by changes in the levels of the ovarian hormones estrogen and progesterone (1). Implantation comprises three stages: Apposition, adhesion and penetration. In the apposition stage, the blastocyst unstably adheres to the endometrial surface (2). Following apposition, the trophoblast and luminal epithelium adhere sufficiently strongly to prevent the displacement of the blastocyst (3). The embryo then invades through the luminal epithelium into the stroma to associate with the maternal vasculature (4).

In rats, implantation occurs at a specific time, during a brief 24-h period 5 days after mating (5,6). Prior to implantation, the endometrium is unresponsive to the blastocyst or to other decidualizing signals. In mammals, implantation is regulated through the normal reproductive cycle and, in numerous mammalian species, may be timed to coincide with conditions that are more favorable for the support of embryonic growth (7). The control of implantation is primarily maternal and achieved through hormone-mediated changes in the expression of adhesion molecules and vessel-related factors.

Osteopontin (OPN) is an adhesion protein (8). Amino acid analysis of OPN has revealed the existence of a conserved cell-binding arginine-glycine-aspartic acid sequence, which has been demonstrated to be involved in adherence to cell surface receptors (9). In the reproductive tract, OPN, which is expressed by secretory-phase endometrial cells, invading trophoblasts, decidual glands and the placenta, is temporally involved in blastocyst invasion and placentation (10,11).

Vascular endothelial growth factor (VEGF) is an endothelial cell-specific mitogen *in vitro* and is known to be the key factor responsible for vasculogenesis and angiogenesis in a variety of models (12). In rodents, VEGF acts in embryo-endometrium interactions by regulating endometrial vascular permeability and endothelial cell proliferation at implantation sites (13,14). Furthermore, VEGF receptor 1 (VEGFR1) and VEGFR2 have been observed in microvessels during the midsecretory period, emphasizing a possible association between the increased microvascular density (MVD) and vascular permeability (15). These studies have determined two basic roles for VEGF in endometrial tissue: a) The regulation of endometrial vascularization and vascular permeability; b) the establishment of a receptive endometrium to support blastocyst implantation and trophoblast invasion.

Assisted reproductive technology (ART) is now available to select high-quality embryos; however, despite such technological advances, implantation rates remain relatively low (16). Uterine receptivity has a key role in the establishment of successful pregnancies, and impaired receptivity may limit the success of ART. Controlled ovarian hyperstimulation (COH) is a frequently used therapeutic strategy in the treatment of infertility; however, the effect of COH on implantation remains controversial (17–19). The use of gonadotropin-releasing hormone agonist (GnRHa) is associated with advanced endometrial maturation of 2–4 days on the day of oocyte retrieval, and no pregnancy occurs when the advancement is >3 days (20). It has additionally been suggested that COH results in supraphysiological levels of estrogen and progesterone, which may impair endometrial receptivity (21).

The use of herbal medicines for the treatment of infertility has been well documented in China for numerous years. In contrast to target-oriented Western medicine, Traditional Chinese Medicine (TCM) uses a holistic and synergistic approach to restore the balance of body energy and to maintain the normal functioning of the body (22,23). According to the theory of TCM, we have developed Zi Dan Yin (ZDY) (Table I) to be used to prepare the endometrium for implantation.

Although there have been marked increased in the understanding of implantation, therapeutic options remain poor. Further studies are required to provide clinical treatment options for patients experiencing implantation failure-related infertility. The incidence of early pregnancy loss during or immediately subsequent to implantation is high (25–40%) (24). Failed implantation is also a major limiting factor in assisted reproduction (25). The aim of the present study was to investigate the effects of COH on implantation biology and pregnancy outcome, and to explore the potential therapeutic role of the TCM Zi Dan Yin (ZDY).

## Materials and methods

### Ethics statement

All of the experimental protocols utilized in the present study were approved by the Ethics Committee of the Beijing University of Chinese Medicine Animal Care and Use Committee (no. 2013-015-A).

### ZDY preparation

All the crude drugs involved in the composition of ZDY were obtained from the Department of Pharmacy, Dongfang Hospital of Beijing University of Chinese Medicine (Beijing, China). The quality of the raw herbs was controlled according to the requirement of the Pharmacopoeia of the People’s Republic of China (26). Aqueous extract of ZDY was prepared in accordance with the following procedure. In brief, nine medicinal materials were mixed in proportion and macerated for 1 h with eight volumes of distilled water and then decocted for 2 h. The cooled extract was subsequently filtered. The extraction procedure was repeated twice. The extracts were then combined and concentrated by boiling to a final volume of 100 ml (4.12 g/ml). This dilution was used in the following preliminary experiments in a range of concentrations from 1.03 to 4.12 g/ml.

### Treatment

Mature, female Sprague Dawley rat virgins aged seven to eight weeks (weight, 200–220 g) were maintained in the Research Centre, Beijing University of Chinese Medicine on a 12-h light/dark regimen with free access to water and a standard diet. The estrous cycle was identified by vaginal smear. Only the female rats with regular cycles were used. Suitable rats were randomly allocated into one of four groups: Control, COH, ZDY and COH + ZDY.

Rats in the COH group were administered 1 ml/100 g distilled water for 12 days and then treated with the GnRHa-long protocol. In brief, the GnRHa (Diphereline™; Ipsen, Boulogne-Billancourt, France) was injected intraperitoneally at 1.5 μg/100 g body weight/day between days 3 and 9 of the estrous cycle. Pregnant mare’s serum gonadotropin (Inner Mongolia Chifeng Bo En Pharmaceutical Co., Ltd., Chifeng, China) was injected intraperitoneally at 5 IU/100 g body weight on the ninth day of the estrous cycle followed by the injection of human chorionic gonadotropin (hCG; Yantai North Pharmaceutical Co., Ltd., Yantai, China) at 10 IU/100 g 28 h later. In the ZDY group, the animals were administered 1 ml ZDY/100 g body weight/day for 12 days followed by saline injections at the same time and volume as the COH group. In the COH + ZDY group, the animals were administered the 1 ml ZDY/100 g daily for 12 days and were then subjected to the same GnRHa-long protocol as the COH group. In the control group, the rats were administered distilled water for 12 days, followed by injections with saline at the same time and volume as the treatments used in the COH group. The female rats were housed overnight with males (1:1) subsequent to being given hCG or saline. Successful mating was checked daily by the presence of vaginal plugs. The morning when the plug was found was designated the day 1 of gestation (D1).

In rats, implantation occurs on the fifth day after coitus. During this specific, temporary period, known as the ‘receptive phase’ and ‘window of implantation’, the endometrium undergoes marked changes in structure and function induced by ovarian steroids, which prepares the endometrium to be receptive to the embryo (5). Thus, to demonstrate the characteristics of the endometrium in a COH rat model during implantation, the rats were sacrificed using 10% chloral hydrate (302-17-0; Solabrio, Beijing, China) prior to implantation (D3 and D4) and during the implantation window (D5) (n=6/day). Whole uteri were collected promptly without excess fat and connective tissue. One-third of each sample was fixed in 4% paraformaldehyde, and the remaining part of each sample was stored at −80°C until protein and mRNA extraction.

### MVD

For the immunohistochemical detection of cluster of differentiation (CD) 34, uteri were fixed for 12 h at 4°C in buffered paraformaldehyde and were then routinely processed for paraffin embedding. The paraffin-fixed tissues were cut into 4-μm sections. Briefly, the slides were dewaxed, rehydrated, blocked and incubated with the primary antibody: Anti-CD34 goat polyclonal antibody (cat. no. sc-1336; Santa Cruz Biotechnology, Inc., Santa Cruz, CA, USA) at a 1:200 dilution overnight at 4°C. Subsequent to rinsing three times with phosphate-buffered saline (PBS), the tissues were incubated with secondary antibody (PV-0003; ZSGB-BIO, Beijing, China) for 25 min, followed by incubation with a 3,3′-diaminobenzidine kit (ZLI-9018; ZSGB-BIO, Beijing, China). As a control, normal PBS was used and the primary antibody was omitted. Finally, the sections were dehydrated, counterstained and mounted for observation.

MVDs were viewed at ×400 magnification (40X objective lens and 10X ocular lens; 0.24 mm^2^/field). Tissue images were captured with a digital camera (Olympus, Inc., Tokyo, Japan). For each section, at least five randomly selected fields were counted to determine the density of the microvessels within the uterus. The number of CD34-positive vessels was quantified using Diagnostic Instruments SPOT imaging software (Diagnostic Instruments, Inc., Sterling Heights, MI, USA). The MVD was calculated as the number of CD34-positive vessels/(40×0.24 mm^2^).

### Western blot analysis

Uterine slices previously frozen at −80°C were incubated and lysed in radioimmunoprecipitation assay lysis buffer (cat. no. C1053; Applygen Technologies, Inc., Beijing, China) supplemented with protease inhibitor cocktail (cat. no. P1265; Applygen Technologies, Inc.). The protein concentration was quantified using a bicinchoninic acid assay (cat. no. P1511; Applygen Technologies, Inc.). Sodium dodecyl sulfate-polyacrylamide gel electrophoresis (SDS-PAGE) using a 10% polyacrylamide gel was performed and the samples were transferred to nitrocellulose membranes (Bio-Rad, Hercules, CA, USA). The membranes were blotted with rabbit polyclonal anti-VEGF (cat. no. ab46154; Abcam, Cambridge, UK) or rabbit polyclonal anti-OPN (cat. no. ab8448; Abcam) primary antibodies at a dilution of 1:2,000 and incubated overnight at 4°C. Following incubation, the membranes were washed three times with Tris-buffered saline/Tween 20 and then incubated with goat anti-rabbit immunoglobulin G secondary antibodies (cat. nos. P1308 and P1309; Applygen Technologies, Inc.) at a dilution of 1:2,000 at room temperature for 1 h. The blots were visualized with Super ECL Plus Detection Reagent (cat. no. P1010; Applygen Technologies, Inc.). The enhanced chemiluminescence signals were detected with Quantity One^®^ software (Bio-Rad). β-actin (cat. no. ab8226; Abcam) was used as the reference protein to validate the amount of protein loaded onto the gel.

### Reverse transcription-quantitative polymerase chain reaction (RT-qPCR)

VEGF and OPN gene expression was validated using qPCR. Total RNA was extracted from the uteri of rats in the control, COH, ZDY and COH + ZDY groups using TRIzol^®^ (Invitrogen Life Technologies, Carlsbad, CA, USA) according to the manufacturer’s instructions. RNA was thawed on ice and quantified spectrophotometrically, and the quality was then assessed using SDS-PAGE. RT was performed with 8 μl total RNA per 20 μl reaction using the standard cDNA Synthesis kit (Takara Bio, Inc., Shiga, Japan). The qPCR primer sequences for the target genes were as follows: GAPDH forward primer, 5′-TGC TGA GTA TGT CGT GGA G-3′ and reverse primer, 5′-GTC TTC TGA GTG GCA GTG AT- 3′ (288 bp); VEGF forward primer, 5′-GGC TCA CTT CCA GAA ACA CG-3′ and reverse primer, 5′-GTG CTC TTG CAG AAT CTA GTG G-3′ (165 bp); OPN forward primer, 5′-GAG GTG ATA GCT TGG CTT ACG G-3′ and reverse primer, 5′-CGC TGG GCA ACT GGG ATG-3′ (154 bp).

For each qPCR reaction, the typical thermal cycling conditions included an initial activation step at 95°C for 5 min, 40 cycles of amplification and a final melting curve (65–95°C). PCR reactions were performed using an ABI Prism^®^ 7700 Sequence Detection system (Applied Biosystems, Foster City, CA, USA). Experiments were carried out in triplicate, and cDNA concentrations were normalized with the GAPDH PCR products. Gene expression was analyzed using the 2^−ΔΔCt^ algorithm.

### Average number of implantation sites and live births

On D10, uteri were collected from each group immediately subsequent to sacrifice (n=6). Whole uteri were collected promptly without excess fat and connective tissue, and the conceptuses were removed from the uteri. The number of implantation sites in the uterine horn was recorded. The average number of implantation sites was calculated as the total number of implantation sites/number of rats. Subsequent to conceiving and bearing newborn rats, another sample of rats (n=6) were used to conceive and bear newborn rats to determine the number of live births from each group. The average number of live births was calculated as the total number of newborn rats/number of rats.

### Statistical analysis

Data are presented as the mean ± standard error of the mean. One-way analysis of variance and least significant difference tests were used with the SPSS 17.0 statistical software package (SPSS, Inc., Chicago, IL, USA). P<0.05 was considered to indicate a statistically significant difference. Graphs of the data were produced using Microsoft Excel software (Microsoft Corp., Redmond, WA, USA).

## Results

### MVD results

A summary of the MVD results is shown in Table II. Through the immunohistochemical analysis, the MVD was found to be significantly increased in the COH group compared with that in the control, ZDY and COH + ZDY groups (P<0.01), with a maximal value on D5. No significant differences were found among the control, ZDY and COH + ZDY groups (P>0.05).

### Western blot analysis results

**Figure 1:**
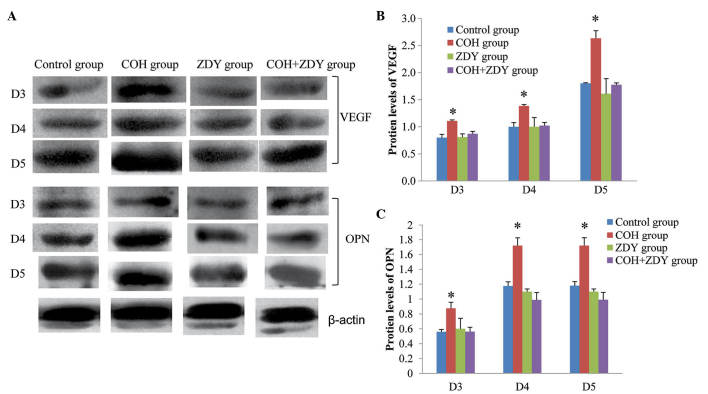
Protein expression of VEGF and OPN. (A) Western blotting was used to measure VEGF and OPN protein levels in COH rats receiving different treatments and controls. (B) VEGF and (C) OPN protein expression in the COH group was increased compared with that in the control, ZDY and COH + ZDY groups. No significant differences were found among the control, ZDY and COH + ZDY groups (n=6). Data are expressed as the mean ± standard error of the mean. ^*^P<0.05 vs. all other groups. VEGF, vascular endothelial growth factor; OPN, osteopontin; COH, controlled ovarian hyperstimulation; ZDY, Zi Dan Yin; D3, day 3 of pregnancy.

The protein expression of endometrial VEGF and OPN during implantation was confirmed by western blotting (Fig. 1). The protein levels of VEGF and OPN were normalized by β-actin. Among the four groups, the VEGF and OPN expression levels in the COH group were significantly higher than those in the other groups on D3, D4 and D5 (P<0.05). No significant differences were found among the control, ZDY and COH + ZDY groups (P>0.05).

### RT-qPCR results

**Figure 2:**
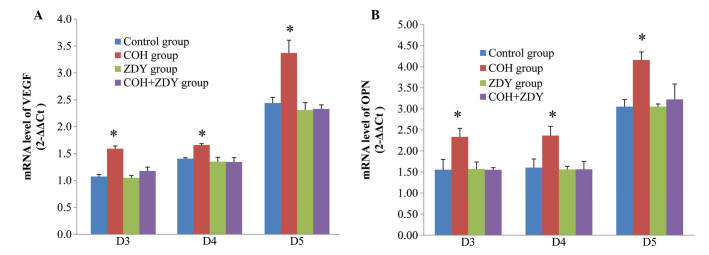
mRNA expression of VEGF and OPN. (A) VEGF and (B) OPN mRNA expression in different groups. VEGF and OPN mRNA expression in the COH group was increased compared with that in the control, ZDY and COH + ZDY groups. No significant differences were found among the control, ZDY and COH + ZDY groups (n=6). Data are expressed as the mean ± standard error of the mean. ^*^P<0.05 vs. all other groups. VEGF, vascular endothelial growth factor; OPN, osteopontin; COH, controlled ovarian hyperstimulation; ZDY, Zi Dan Yin; D3, day 3 of pregnancy.

To demonstrate whether the VEGF and OPN mRNA expression results were consistent with the results of protein expression, RT-qPCR analysis was used (Fig. 2). VEGF and OPN mRNA were found to be expressed in the endometrium of the rats during the implantation window. Compared with the control, ZDY and COH + ZDY groups, the VEGF and OPN mRNA expression levels, normalized by GAPDH, in the COH group were significantly increased (P<0.05). During the implantation window, no significant differences were found among the control, ZDY and COH + ZDY groups (P>0.05).

### Measurements of the number of implantation sites and live births

The effects of COH and/or ZDY treatment on the number of implantation sites and live births are summarized in Table III. The number of implantation sites and live births in the COH group was significantly lower than that in the other groups. No significant differences were found among the control, ZDY and COH + ZDY groups.

## Discussion

The early stages of embryo implantation in rats are characterized by two endometrial vascular events: A localized increase in endometrial vascular permeability, which is the earliest indicator of the implantation, and increased endothelial cell proliferation (13). VEGF exhibits a potent vascular permeability-inducing action and has been revealed to be the factor responsible for implantation-related increases in vascular permeability in the rat endometrium. Furthermore, the role of VEGF in vascular beds has been demonstrated in other mammalian species, including humans, pigs and rabbits (27,28). A previous study showed that endothelial cell proliferation was increased from the third day of pregnancy and remained elevated throughout the entire endometrium up to the fifth day of pregnancy (29). The present study indicated that VEGF can be found in the rat endometrium on D3, D4 and D5.

One of the commonest methods of indirectly assessing vasculogenic and angiogenic activity is to examine and compare the net gain or loss in the number of microvessels between different tissues. This is most frequently used to count the number of vessel cross-sections or profiles present in a specified area of a histological section. Following the invasion of the maternal endometrium, embryonic development is characterized by a marked growth of blood vessels occurring concurrently with decidualization and the development of vascular membranes (30). Previous studies have demonstrated the presence of VEGF mRNA in trophoblast cells in rats (31) and mice (32). These findings suggest that VEGF may be a key factor in the induction of vascular growth in the decidua and vascular membranes; however, there is little knowledge concerning the direct association between the MVD and VEGF expression. To the best of our knowledge, this study is the first to measure the MVD, as well as VEGF expression, in a COH rat model. The findings showed that the changes in MVD and VEGF expression are consistent in the rat endometrium throughout implantation. Furthermore, OPN showed the same expression trend at the same time. We suggest the existence of an association between the MVD and the levels of VEGF and OPN.

Implantation is a complex process involving proliferation and tissue remodeling. Adhesion molecules and cytokines have been suggested to play key roles in this process (33), and OPN has the capacity to act as both a cytokine and an adhesion molecule. A previous study has shown that OPN expression is increased in the secretory-phase endometrium and decidua of pregnant females (34). Furthermore, microarray studies have consistently found a 4.9- to 20-fold upregulation of OPN during this period in humans (33–38). In mice, OPN induces blastocysts to activate their adhesion competence through the formation of integrin adhesion complexes at the trophectoderm cell surface (39). By contrast, OPN-null mice remain fertile (40,41). In the present study, OPN protein levels were low on D3 and D4 and markedly increased on D5. OPN mRNA levels were low on D3, and markedly increased on D4 and D5. OPN expression therefore increased throughout the implantation process. It has been suggested that angiogenesis and vascular remodeling is facilitated by OPN and α_v_β_3_ (42). This is one possible explanation for the increased OPN expression associated with increased MVD.

COH was observed to have a negative effect on the endometrium, as the COH rat model exhibited a significant higher MVD and VEGF and OPN expression compared with the control group rats. Previous studies have reported increased pre-implantation mortality following superovulation in mice (43,44), hamsters (45) and rats (46). Furthermore, impaired development and reduced implantation rates were found with embryos from superovulated donors, although the weight of the live fetuses obtained following transfer from superovulated donors was not significantly different from that of control embryos; this may suggest that superovulation has certain negative effects even on viable embryos (47). Failed implantation is a significant limiting factor in assisted reproduction (25). Since ovarian stimulation generates a cascade of hormonal and physiological events, the embryos mature in an environment that exhibits differences from the environment in which embryos mature naturally (48). Furthermore, variations may also be apparent in the timing of ovulation and implantation (49). The relative contribution of the endometrium to the rate of successful reception is not currently known, and no accepted criteria exist for the evaluation of endometrial implantation; however, preparations of the endometrium-produced cytokines may mediate these precisely defined morphological changes (21,50). In the present study, the expression of VEGF and OPN in the COH group was higher than that in the control, ZDY and COH + ZDY groups, indicating that COH may impair the synchronization of embryonic development and endometrial maturation; however, the adverse effects of COH cannot only be attributed to asynchrony, as an early study showed that superovulation following synchronization also resulted in increased embryonic loss (43).

In the present study, ZDY suppressed the COH-induced increases in MVD and VEGF and OPN expression in rats. No significant differences were found between the control and ZDY groups. These results therefore suggest that a TCM such as ZDY could prove to be of clinical use in patients with impaired endometrial receptivity subsequent to COH; however, TCM cannot translate the normal state into a super-normal state, which accounts for a lack of significant difference between the control and ZDY groups. A further clinical study is required to confirm the proposed approach in this aspect.

In combination, the present results indicated that COH treatment increased the expression of MVD, VEGF and OPN during implantation. The study showed that VEGF and OPN may play important roles during implantation, and that an association exists between changes in the MVD and VEGF and OPN expression. In addition to the molecular and structural markers of endometrial receptivity, MVD could be an alternative approach to identify this period of receptivity in rats. The present study also revealed that high levels of VEGF and OPN were apparent subsequent to COH treatment. These findings confirmed the adverse effects of COH with GnRHa on implantation, which have been documented in previous studies (47,51). ZDY markedly restored the endometrial MVD and VEGF and OPN expression during implantation in the COH rat model. The present study provides novel insight into TCM approaches for infertility treatment and ART.

## Figures and Tables

**Table I:** Composition of Zi Dan Yin.

Components	Ratio
Sheng Di [*Rehmannia glutinosa* (Gaertn.) Libosch., root)]	15
Dan Shen (*Salviae miltiorrhizae* Bge., root)	10
Dang gui [*Angelica sinensis* (Oliv.) Diels., root]	12
Chuan Duan (*Dipsacus asperoides* C.Y. Cheng et T.M. Ai., root)	15
Du Zhong (*Eucommia ulmoides* Oliv., cortex)	12
Shan Yao (*Dioscorea opposita* Thunb., rhizome)	15
Mei Gui-hua (*Rosa rugosa* Thunb., flower)	6
Chuan Xiong (*Ligusticum Chuanxiong* Hort., rhizome)	6
Yi Yi-ren [*Coix lacryma-jobi* L. var. ma-yuen (Roman.) Stapf., seed]	12

**Table II:** Microvascular density.

Group	D3 (n)	D4 (n)	D5 (n)
Control	1.79±0.06	2.88±0.19	3.56±0.11
COH	2.24±0.09^a^	3.91±0.07^a^	4.34±0.11^a^
ZDY	1.86±0.03	2.88±0.09	3.34±0.10
COH + ZDY	1.74±0.07	2.97±0.08	3.32±0.09

The density of microvessels within the uterus was determined on D3, D4 and D5. Data are presented as the mean ± standard error of the mean (n=6 in each group).

a P<0.05 vs. all other groups. No significant differences were found among the control, ZDY and COH + ZDY groups.

The groups were established as follows: COH, GnRHa-long protocol-stimulated rats; ZDY, rats that received ZDY treatment; COH + ZDY, GnRHa long protocol-stimulated rats that received ZDY treatment. COH, controlled ovarian hyperstimulation; ZDY, Zi Dan Yin; D3, day 3 of pregnancy; GnRHa, gonadotrophin-releasing hormone agonist.

**Table III:** Number of implantation sites and live births.

Parameter	Control	COH	ZDY	COH + ZDY
Implantation sites (n)	10.33±0.84	3.67±0.49^a^	10.00±0.73	8.67±0.33
Live births (n)	11.00±0.63	4.33±0.42^a^	10.67±0.71	9.33±0.61

Data are presented as the mean ± standard error of the mean (n=6 in each group).

a P<0.05 vs. all other groups.

No significant differences were found among the control, ZDY and COH + ZDY groups. The groups were established as follows: COH, GnRHa long protocol-stimulated rats; ZDY, rats that received ZDY treatment; COH + ZDY, GnRHa-long protocol-stimulated rats that received ZDY treatment.

COH, controlled ovarian hyperstimulation; ZDY, Zi Dan Yin; D3, day 3 of pregnancy; GnRHA, gonadotrophin-releasing hormone agonist.
